# Communication Intervention in South Africa: Advocating for the Listening and Spoken Language Approach

**DOI:** 10.4102/sajcd.v71i1.1071

**Published:** 2024-10-28

**Authors:** Aisha Casoojee

**Affiliations:** 1 Department of Audiology, Faculty of Humanities, University of the Witwatersrand, Johannesburg, South Africa

**Keywords:** early intervention, EHDI, Listening and Spoken Language, communication outcomes, hearing impairment, contextual healthcare, South Africa, speech-language therapy, audiology, family-centred care

## Abstract

Adhering to Early Hearing Detection and Intervention (EHDI) guidelines is critical for the timely identification and management of infants with hearing impairment. Early intervention specifically focusses on family-centred communication interventions, of which the Listening and Spoken Language–South Africa (LSL-SA) approach is available for children with hearing impairment in South Africa. Studies have evidenced significant benefits for children with hearing impairment enrolled in LSL-SA compared to those enrolled in Traditional Speech-Language Therapy (TSLT), that is, age-equivalent communication outcomes, shorter duration of therapy until discharge, meritorious academic performance and integration into a hearing society. Despite these positive outcomes related to the LSL-SA approach, significant contextual barriers hinder effective implementation nationwide.

**Contribution:**

The successful implementation of EHDI guidelines in South Africa hinges on overcoming barriers through tailored healthcare strategies and contextualised delivery. Adapting frameworks such as LSL-SA to fit the local context is crucial for advancing equitable access to EHDI services, positively impacting children with hearing impairment and their families. This article highlights the systemic challenges in South Africa in optimising resources by adopting linguistically appropriate and culturally responsive early intervention approaches to champion hearing healthcare initiatives. Effective collaboration among diverse stakeholders is essential for enhancing the uptake of EHDI guidelines and translating policy into impactful communication interventions. Implementing the core principles of the LSL-SA approach will not only ensure access to this specialised service for suitable candidates but may also alleviate specific barriers to EHDI implementation within TSLT approaches.

In South Africa, a low-middle-income country (LMIC), children with hearing impairment face unique challenges in accessing health services crucial for their development and societal integration (Bush et al., 2017; World Health Organization [WHO], 2016). The Health Professions Council of South Africa’s (HPCSA) Professional Board for Speech, Language, and Hearing Professions, in alignment with the Joint Committee on Infant Hearing’s (JCIH) 2007 position statement, drafted an Early Hearing Detection and Intervention (EHDI) guideline (HPCSA, 2018). This guideline proposes to identify, diagnose and provide intervention for children with hearing impairment at the earliest stages of infancy, enabling effective communication and promoting their full developmental potential (HPCSA, 2018). According to the HPCSA, South African speech-language therapists (SLTs) and audiologists play a crucial role in achieving developmental goals by offering fair, accessible and equitable early childhood development services that account for contextual realities. They are also responsible for providing specialised rehabilitation services to children with hearing impairment and their families, as outlined in the HPCSA EHDI Guideline (HPCSA, 2018; Khoza-Shangase, 2021b; Pascoe & Singh, 2023; Pillay et al., 2020). The choice of communication intervention approach plays a pivotal role in determining speech-language acquisition and overall educational outcomes of children with hearing impairment (Casoojee et al., 2024; Casoojee & Khoza-Shangase, 2022; Maluleke et al., 2021a). The current landscape of early communication interventions in South Africa for children with hearing impairment and their families employs one of the following two approaches:

1. Traditional Speech-Language Therapy (TSLT) is a more cost-effective intervention approach, given the challenges within South Africa (Casoojee et al., 2021). It encompasses a range of communication methods, from visual-gestural approaches such as sign language, total communication and cued speech to auditory methods involving natural gestures, speechreading and visual cues combined with amplification (Casoojee et al., 2021). Sign language acquisition follows a developmental trajectory analogous to spoken language, provided children with hearing impairment receive consistent input from fluent signers from birth (Lillo-Martin & Henner, 2021). However, the acquisition context varies significantly: while deaf children with deaf signing parents have an optimal linguistic environment for sign language acquisition, more than 90% of deaf children are born to hearing, non-signing families (NIH, 2015). In these cases, a lack of exposure to spoken language because of hearing impairment and insufficient proficiency in sign language among parents result in delayed language acquisition (Hall et al., 2017).
2. Listening and Spoken Language-South Africa (LSL-SA) is a postgraduate diploma offered through the University of Stellenbosch, which serves as an additional licensing course following an undergraduate qualification. It is based on the LSLS Certified Auditory-Verbal Therapist (LSLS Cert. AVT®) qualification by the AG Bell Academy. The LSL-SA emphasises auditory-verbal techniques aimed at maximising a child’s ability to listen, understand spoken language and speak (Estabrooks, 2020). Seven of the 10 LSL principles involve parents training their children to integrate listening skills into communication and social development. Blose and Joseph (2018) found that one or two hours of weekly intervention were insufficient to address early auditory and language deprivation in children with hearing impairment, which highlights the importance of empowering South African parents to become primary facilitators of their child’s speech and language development (Casoojee et al., 2024a; Khoza-Shangase, 2022). The LSL principles further advocate for early diagnosis of hearing impairment and prompt audiological management, utilising advanced hearing technology for maximum auditory stimulation benefits. These principles bind LSL interventionists (AG Bell Academy, 2018), leveraging natural developmental listening, language, speech and cognition patterns to promote effective communication. An LSL approach aims to facilitate the educational and social integration of children with hearing impairment into mainstream schools (AG Bell Academy, 2018).

A global body of evidence supports the effectiveness of the LSL approach (Auditory Verbal UK, 2023). Emphasising natural language development and cognitive growth through structured therapy activities, the LSL approach involves parents as partners in fostering listening and language skills during daily routines and play (Chowdhry, 2010). Research demonstrates that children with hearing impairment in LSL programmes attain spoken language commensurate with their normal hearing peers, irrespective of hearing technology, and make similar progress in listening skills, reading, mathematics and self-esteem (Cupples et al., 2017; First Voice, 2023; Fulcher et al., 2012; Hitchins & Hogan, 2018; Mortazavi & Mortazavi, 2017; Noel et al., 2023; Percy-Smith et al., 2017). Research further indicates that children with hearing impairment enrolled in LSL programmes achieve comparable academic progress, societal interaction and social role fulfilment as their normal hearing peers, promoting better social integration within a predominantly hearing society (Constantinescu-Sharpe et al., 2017).

The rationale for advocating the broader adoption and integration of the LSL approach within the South African context is justified by empirical evidence, its adaptability to local challenges and the potential to improve access and equity in services for children with hearing impairment. This article further explores how the LSL approach can be practically applied amid contextual challenges such as access to services, service delivery issues, continuity of care, language and cultural barriers, and socio-economic disparities (Khoza-Shangase, 2021b).

## Overcoming barriers and enhancing LSL-SA implementation for improved outcomes

Effective healthcare must integrate contextual considerations to optimise intervention outcomes, ensure intervention relevance and address sustainability challenges to achieve widespread impact (Coles et al., 2020). Adapting approaches such as LSL-SA to meet the specific needs of children with hearing impairment is crucial. This involves fostering collaboration among early interventionists, SLTs, audiologists, parents, educators and government stakeholders. By integrating contextualised healthcare practices within communication interventions such as the LSL approach, South Africa can significantly enhance the quality of care, support holistic development and promote societal integration for children with hearing impairment (Hlayisi et al., 2024; Khoza-Shangase & Mophosho, 2018). Leveraging effective, collaborative strategies is key to overcoming the challenges presented by the high prevalence of child hearing impairment in South Africa and achieving successful implementation of the LSL-SA approach.

In 2021, South Africa recorded nearly 1 million births (Stats SA, 2021), with an estimated 3 to 6 out of every 1000 babies born with hearing impairment (Khoza-Shangase, 2021a). Congenital hearing impairment is particularly prevalent in the public healthcare sector (Khoza-Shangase, 2021b), which serves 82.6% of the population (Stats SA, 2016). This sector faces significant strain, exacerbated by a shortage of skilled health professionals, including SLTs and audiologists (Matthews & Van Wyk, 2018). Only 22.0% of SLTs and audiologists are employed in public healthcare, highlighting a critical shortfall of 2800 professionals and the need for a 300.0% growth rate by 2030 to meet healthcare demands (Pillay et al., 2020). In addition, South Africa grapples with the limited availability of LSL-SA interventionists, with only 51 LSL-SA therapists and six qualified LSLS Cert.AVT^®^ therapists nationwide, predominantly concentrated in the private healthcare sector located in urban areas (Casoojee et al., 2024b; South African Cochlear Implant Group, 2022). This disparity underscores challenges in public–private healthcare sectors and urban–rural service provision in meeting the needs of children with hearing impairment. Addressing these disparities necessitates a concerted effort to (1) increase the number of trained LSL-SA and LSLS Cert.AVT^®^ qualified therapists across South Africa, (2) acquire funding to support the development and delivery of LSL-SA programmes and investment in research, (3) coordinated management, (4) community and stakeholder engagement, (5) infrastructure development (access to facilities and technology integration), and (6) evaluation and feedback mechanisms.

The HPCSA EHDI Guidelines (2018) emphasise the roles of SLTs and audiologists in promptly selecting, fitting and monitoring amplification devices. The guidelines recommend that SLTs and audiologists with expertise in cochlear implants participate in assessing candidacy for such devices (HPCSA, 2018). The LSL-SA approach rests heavily on a child’s access to the acoustic features of speech provided by hearing aids and cochlear implantation (Casoojee et al., 2021; Dettman et al., 2016). Addressing the accessibility and cost issues associated with these devices (Casoojee et al., 2021; Khoza-Shangase, 2021b) is crucial to making LSL-SA a viable option within the South African public healthcare sector. Solutions could include increasing the availability of hearing aids and cochlear implants through public healthcare programmes, subsidising costs to make them more affordable and advocating government funding and support. By improving access to these amplification devices, South Africa can enhance the implementation of the LSL-SA approach, ensuring children with hearing impairment benefit from its proven effectiveness.

Children with disabilities in South Africa face significant barriers to healthcare and education because of limited access to appropriate interventions, which diminishes their potential for success (Kuper & Hancock, 2020). Despite efforts by the South African Department of Basic Education to create an inclusive education system, progress remains slow (Khumalo & Hodgson, 2017). Research by Casoojee et al. (2024) shows that children with hearing impairment enrolled in LSL-SA achieve age-equivalent language outcomes and are more likely to qualify for mainstream schooling compared to those in TSLT. The study, which matched learners by age and classification of hearing impairment, excluded those with unilateral profound hearing impairment or bilateral profound hearing impairment with significant comorbidities, highlights that 64% of the TSLT cohort and 77% of the LSL-SA cohort wore cochlear implants, while 36% of the TSLT cohort and 23% of the LSL-SA cohort used hearing aids. These findings underscore the need to address accessibility to LSL-SA and cost issues related to hearing devices to enhance the viability of LSL-SA. Supporting LSL-SA aligns with the National Development Plan 2030’s goals for early intervention, inclusive education, and continuity of care (Casoojee et al., 2024b).

Children with hearing impairment enrolled in LSL-SA were enrolled in therapy for a shorter duration until discharge than those enrolled in TSLT. This is a significant finding, particularly in LMICs, offering a possible reduction in the global burden of disease caused by hearing impairment in this context (Casoojee et al., 2024). In a country with limited resources, constrained socio-economic support by the state, as well as capacity versus demand of SLTs and audiologists and workforce challenges (Khoza-Shangase, 2022; Khoza-Shangase et al., 2021; Pillay et al., 2020), these positive outcomes of LSL-SA are financially, and quality of life justified to be pursued. Research attests that implementing an LSL-SA intervention approach is key to improving communication outcomes for children with hearing impairment (Casoojee et al., 2024. By reducing therapy duration and improving communication outcomes, LSL-SA addresses both cost and quality-of-life concerns effectively. Leveraging the benefits of LSL-SA, includes (1) advocating for increased funding and support for LSL-SA programmes, (2) expanding training opportunities for SLTs and audiologists, and (3) ensuring that LSL-SA is integrated into public healthcare policies. By focussing on these areas, South Africa can enhance the implementation of LSL-SA, making it a viable and impactful solution for improving the lives of children with hearing impairment.

Despite the above-stated positive findings associated with the LSL-SA approach, a study by Casoojee et al. (2024a) reveals that its effectiveness is compromised by a lack of culturally sensitive and linguistically appropriate resources. This is particularly important in South Africa, where linguistic and cultural diversity is critical to effective clinical service provision (HPCSA, 2019; Khoza-Shangase & Mophosho, 2021; Maluleke & Khoza-Shangase, 2022; Mophosho et al., 2022). Therefore, LSL-SA interventionists must prioritise the development and implementation of resources that are both culturally and linguistically responsive to meet the needs of diverse communities (Taylor, 2016). In the South African context, providing LSL-SA intervention in a language disparate from the home language of children with hearing impairment and their families does not respond to their needs, resulting in poorer communication outcomes (Al Shamsi et al., 2020; Khoza-Shangase & Kalenga, 2023).

A significant barrier to effective communication arises when healthcare providers, especially SLTs and audiologists, do not share a first language with the population they serve (Khoza-Shangase & Kanji, 2021; Khoza-Shangase & Mophosho, 2018; Pillay & Kathard, 2015). In South Africa’s multilingual context, isiZulu is the most spoken language, followed by isiXhosa, Afrikaans and English (Dauncey, 2023). Only 5% of SLTs and audiologists in South Africa speak an African language as their mother tongue, highlighting workforce demographic incongruities (Khoza-Shangase & Mophosho, 2018). English is the Lingua Franca in South Africa, posing challenges in meeting diverse family needs, particularly in implementing the LSL-SA approach. Furthermore, the population group of SLTs and audiologists in South Africa are classified as white (59.7%), followed by Indian (15.4%), black (15.2%), mixed race (4.7%) and an unidentified population group of these professionals of 4.7% (Pillay et al., 2020). Conversely, risk profile studies of childhood hearing impairment in South Africa indicate that the most prevalent languages and cultures are first-language speakers of an African language as opposed to English and Afrikaans (35%) (Ehlert & Coetzer, 2020; Kuschke et al., 2020; Louw et al., 2018; Swanepoel et al., 2013). To address these disparities and enhance the effectiveness of the LSL-SA approach, several culturally sensitive modifications are required, including (1) the development of multilingual resources to bridge the communication gap and ensure interventions are accessible and relevant to diverse families, (2) cultural sensitivity training of SLTs and audiologists to effectively connect with the families of children with hearing impairment, (3) involving parents as partners to ensure the intervention is more readily accepted and implemented, (4) training of LSL-SA interventionists from diverse linguistic backgrounds (Casoojee et al. 2024a). By integrating these modifications, the LSL-SA approach can become more effective and inclusive, mitigating these challenges significantly (Casoojee et al., 2024b; Maluleke et al., 2021b; Maluleke, 2024; Matthews & Van Wyk, 2018).

To improve access to the LSL-SA communication intervention approach in South Africa, it is imperative that SLTs and audiologists address the diverse needs of children with hearing impairment and their families, employing pragmatic, linguistically appropriate, and culturally sensitive resources and skills. Implementing LSL-SA resources that are both linguistically and culturally congruent can foster strong partnerships with parents and caregivers, significantly improving therapy outcomes (Casoojee et al., 2024a; Khoza-Shangase, 2019; Maluleke, 2024; Maluleke et al., 2023). The Patients’ Rights Charter in South Africa advocates the importance of equitable healthcare access and effective communication between providers and patients to achieve optimal health outcomes (HPCSA, 2016; Jardien-Baboo et al., 2019; Khan et al., 2023). By integrating these approaches, the LSL-SA method can be more responsive to the South African context, ultimately enhancing the quality of care for children with hearing impairment.

## Conclusion

This article advocates for access to the LSL-SA intervention approach, underscoring its significant benefits for children with hearing impairment who meet the candidacy criteria. By leveraging the contextual research evidence (Casoojee et al. 2024a, 2024b; Casoojee et al., 2024; Taylor, 2016), it emphasises the urgent need for equitable and effective interventions in South Africa. The LSL-SA approach is particularly compelling, as it addresses the unique needs of children with hearing impairment and their families achieving age-appropriate communication outcomes, academic achievements and societal inclusion (Mirzoev & Kane, 2017; Weiner, 2021). Notably, children with hearing impairment enrolled in LSL-SA were recommended for mainstream schooling based on their communication outcomes after early intervention, suggesting a positive step towards an inclusive society and potentially reducing the cost of hearing impairment within a resource-constrained LMIC milieu (Casoojee et al., 2024).

Despite the challenges posed by limited resources and workforce constraints, the potential benefits of LSL-SA transcend these obstacles. This article calls for the engagement of various stakeholders – academic institutions, healthcare providers, SLTs, audiologists, government officials and professional bodies – to champion policies supporting LSL-SA implementation. Key actions include securing funding, reframing training programmes, capacity building among SLTs, audiologists and educators, and launching public awareness campaigns to ensure the widespread adoption of LSL-SA and its benefits for suitable candidates among children with hearing impairment and their families in South Africa.

Adapting LSL-SA to fit the South African context is crucial to establishing contextually relevant care that meets the unique needs of children with hearing impairment and their families. By upholding patient rights to timely and effective communication intervention (WHO, 2024), South Africa has the opportunity to set a precedent for inclusive and responsive healthcare practices in LMICs. This proactive stance not only addresses immediate needs but also paves the way for a more equitable, empowered and inclusive society.
